# Modeling moisture sorption isotherms of milk powders at ambient and elevated temperatures using the dynamic dewpoint isotherm (DDI) method

**DOI:** 10.3168/jdsc.2024-0683

**Published:** 2025-06-03

**Authors:** Erika Kadas, Abass Oduola, Peter M. Rubinelli, Griffiths G. Atungulu, Jennifer C. Acuff

**Affiliations:** Department of Food Science, Center for Food Safety, University of Arkansas System Division of Agriculture, Fayetteville, AR 72704

## Abstract

The water activity of milk powders is a critical parameter for predicting quality and safety, but some retailers in the supply chain may be limited to measuring moisture content, which can be easier and more affordable. Moisture sorption isotherms relate moisture content to the corresponding water activity. In this study, moisture adsorption and desorption isotherms were determined for nonfat dry milk (NFDM) and milk protein concentrate (MPC-85) powder samples at ambient and elevated temperatures via the modernized dynamic dewpoint isotherm (DDI) method. Previously cited models (with coefficients determined through linear regression) were also fit to the data for predictive applications of determining shelf life and the potential changes to quality that thermal treatments or elevated storage temperatures could cause. The adsorption isotherms of NFDM were type II, whereas MPC-85 adsorption isotherms were type III. Both NFDM and MPC-85 exhibited type III desorption isotherms, likely due to crystalline components. Temperature significantly affected the adsorption and desorption isotherms of both NFDM and MPC-85. Hysteresis was observed between the adsorption and desorption isotherms for both powders at all temperatures, but the magnitude was greater for MPC-85. The modified Oswin, modified Chung-Pfost, and Guggenheim, Anderson, de Boer models best described NFDM and MPC-85 adsorption and desorption to different degrees, highlighting that treatment and storage of the powders are influenced by both composition and temperature. These findings provide tools for prediction and assessment of quality of 2 vastly different milk powders, which inform handling, processing, and storing milk powders with different protein and lactose concentrations.

## Highlights

• The DDI method provided adsorption and desorption isotherms of milk powders.
• Three temperatures were used to represent different storage and treatment conditions.
• Temperature significantly impacted the isotherms and model coefficients.

## Graphical Abstract

**Figure:**
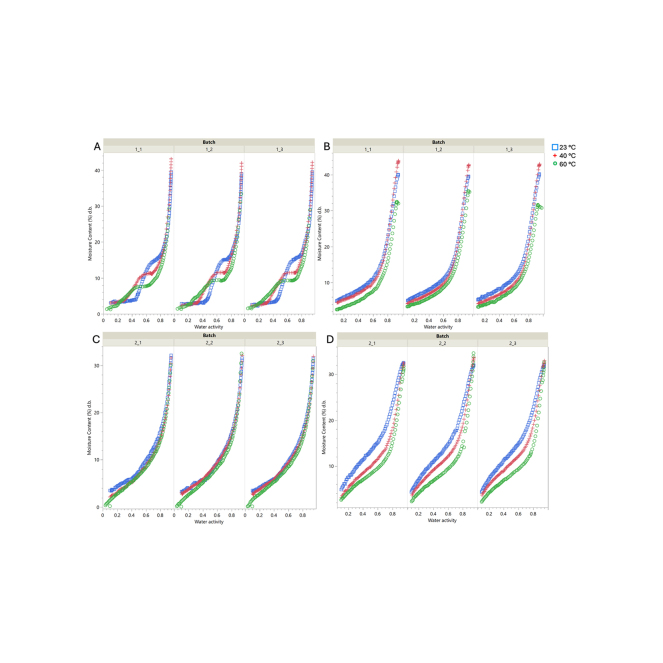
**Summary:** This study demonstrates the importance of water activity in predicting milk powder characteristics. Moisture content is easier to measure but is limited in overall application. The combination of the 2 measurements through moisture sorption isotherms offers useful insights. Using the dynamic dewpoint isotherm (DDI) method, moisture adsorption and desorption isotherms for nonfat dry milk (NFDM) and milk protein concentrate (MPC-85) at different temperatures were examined. The NFDM showed sigmoidal (type II) adsorption isotherms, whereas NFDM desorption and MPC-85 isotherms displayed J-shaped (type III) isotherms. Temperature significantly impacted isotherms, and hysteresis was more pronounced in MPC-85. Models like Oswin and Chung-Pfost effectively described these moisture interactions and could be used for predictive estimations of shelf life and other quality changes. The findings demonstrate how composition and temperature influence the quality of milk powders, aiding manufacturers in handling, processing, and storage.

Moisture sorption isotherms are useful in the food industry for optimization of water content for food safety, quality, and storage stability (Schmidt and Lee, 2012; Hawa et al., 2020; Rodríguez-Martínez et al., 2020; Liu et al., 2022), and optimization of packaging requirements based on sorption properties of products (Suppakul et al., 2013). These isotherms relate the water activity (**a_w_**) of foods and corresponding moisture content of the food, at a given temperature. The availability of water in the food system affects both the product stability and the microbiological safety of the food. As it pertains to food, a_w_ is the ratio of the vapor pressure of a food when in equilibrium with the surrounding environment, to the vapor pressure of pure water under identical conditions (i.e., temperature and pressure) and is a metric of the available water (Schmidt and Lee, 2012; US Food and Drug Administration, 2014). Moisture content is the total amount of water in a food, bound and unbound. Technically, moisture content is described as the mass of water in a food per mass of dry food (Schmidt and Lee, 2012). The relationship between moisture content and a_w_ in a food can be depicted through a moisture sorption isotherm of a food sample. The moisture sorption isotherm is the moisture content of a food plotted as a function of the a_w_ of the food (i.e., food relative humidity) at a constant temperature, which may or may not be linear (Schmidt and Lee, 2012).

The present study used the dynamic dewpoint isotherm (**DDI**) method, but, historically, the 2 most well-known and used methods have been the saturated salt slurry method and the dynamic vapor sorption (**DVS**) isotherm method. These methods, although useful, have their limitations. The saturated salt slurry method involves placing the food over salt slurries that are at a temperature-dependent a_w_, contained in a closed container with desiccators, and then waiting for the food to reach the same a_w_ as the salt slurry via a minimal weight change. Although advantageous because this method generates precise a_w_ values, this method requires extensive time and labor due to having to wait for the sample to equilibrate, resulting in potential mold growth during long equilibration periods at a_w_ ≥0.70 (Yu et al., 2008; Schmidt and Lee, 2012). The DVS method, in contrast, is a modern and automated version of the saturated salt slurry method. Many modern moisture sorption instruments, such as the vapor sorption analyzer (**VSA**) used in this study, are built to automatically change the ratio of dry to wet gas to achieve different relative humidity levels and thereby change a_w_ in a dynamic stepwise manner (Carter and Fontana, 2016). Although the DVS method is much more modernized than the saturated salt slurry method, it can still take up to weeks and months to generate isotherms due to the sample having to reach gravimetric equilibrium before proceeding to the next relative humidity level (Mettler Toledo., 2018). The present study chose to develop the isotherms of the dried milk samples using the updated DDI method, which has not previously been done.

For the present study, the comprehensive DDI method was chosen due to the significantly faster generation of isotherm curves, as well as higher-resolution isotherms with hundreds of data points rather than only 5 or 6, by using a VSA (Aqualab VSA, Decagon Devices Inc., Pullman, WA). The high-resolution isotherms with more data points provide better prediction capabilities with more exact model coefficients. Furthermore, the multiple replications performed in the present study also help reduce or remove random and practical errors. The DDI method directly measures a_w_ with a chilled mirror dewpoint sensor while weight changes in the sample are tracked. For adsorption, the air is saturated with water before entering the sample chamber. For desorption, the air is passed through a desiccator before being able to enter the sample chamber. Once a small change in a_w_ occurs (usually around 0.015), the airflow is immediately halted, and a snapshot of the sorption is captured via a direct measurement of sample a_w_ and weight, which is then converted to moisture content (Schmidt and Lee, 2012). This repeated process produces over 100 data points over a wide range of a_w_ (0.030–0.950) and temperature (15°C–60°C) values (Schmidt and Lee, 2012; Meter Group, 2022).

Moisture sorption isotherms can vary widely between products, even among similar milk powders, due to factors like protein content, lactose crystallization, and temperature (Bronlund and Paterson, 2004; Foster et al., 2005; Shrestha et al., 2007). A one-size-fits-all approach is unsuitable for predicting shelf life and stability. For instance, milk protein concentrate (**MPC**) has nearly twice the protein of nonfat dry milk (**NFDM**), whereas NFDM has more lactose—both affecting isotherms. Foster et al. (2005) observed no adsorption differences at <37°C, but differences emerged at 50°C. Variations in treatment and storage affect shelf life, as seen in adsorption and desorption trends. Murrieta-Pazos et al. (2011) also found that composition and particle size affect water diffusivity in milk powders. Both DVS and salt slurry methods have shown consistent relationships between moisture content and water activity.

The objective of the present study was to determine the moisture sorption isotherms of 2 milk powders that have vastly different compositions, have not been compared using the DDI method, and do not have established isotherms or prediction models at the chosen range of temperatures: NFDM and a MPC with 85% protein (**MPC-85**). Moisture content is a universal metric used in food production, so using the moisture sorption isotherms to indirectly describe the a_w_ will aid processors in making judgments about the properties of their food. Dairy powder producers or retailers may be more likely or better able to determine the moisture content of a product or lot. The moisture sorption isotherms produced in this study will assist the dairy industry with correlating the moisture content with a_w_, which will have further applications for the stability and quality of the product during and after treatment and storage at different temperatures. Three replications of proximate analyses of NFDM (Mars Inc., McLean, VA) and MPC-85 (Dairy Farmers of America, Kansas City, MO) were conducted before isotherm experiments according to official Association of Official Agricultural Chemists methods for moisture content, ash, protein, fat, and carbohydrate levels (± SD; AOAC 927.05, 930.30, 932.06, 930.29; AOAC International, 2023). Three different shipments from the same supplier (representing 3 different lots) of each product were sent for analyses, which were henceforth considered “batches.” The moisture contents of the NFDM and MPC-85 powders were 3.25% ± 0.30% and 5.59% ± 0.03%, respectively. The protein contents of the NFDM and MPC-85 powders were 37.59% ± 0.65% and 86.38% ± 0.17%, respectively, when a factor or 6.38 was used to convert nitrogen to protein. The fat contents of the NFDM and MPC-85 powders were 0.52% ± 0.09% and 1.23% ± 0.00%, respectively. The sugar contents of the NFDM and MPC-85 powders were 51.70% ± 0.69% and 3.01% ± 0.00%, respectively. The ash contents of the NFDM and MPC-85 powders were 7.86% ± 0.13% and 6.53% ± 0.01%, respectively. The different lactose and protein contents specifically have implication on the crystallization and caking of powders, increasing the significance of prediction models from the isotherms.

Moisture sorption isotherms of NFDM and MPC-85 were determined using the DDI method. A VSA (Meter Group Inc., Pullman, WA) was used to determine the isotherms at 3 temperatures (23°C, 40°C, and 60°C), chosen to represent typical storage or potential thermal treatment temperatures. Before the experiment, appropriate desiccant and water levels in the VSA were confirmed, and the moisture analysis toolkit software was configured for the experimental parameters (DDI method, 10%–95% equilibrium relative humidity [**ERH**] range, temperature, 1% resolution, and the airflow rate of 100 mL/min). The milk powder samples were sealed and held at refrigeration temperatures (~4°C) until use, at which point, they were equilibrated to room temperature (~23°C) for the experiment while in their closed containers. Three replications at each temperature were performed for each powder. For the experiment, ~1.0 g of powder was placed in a stainless-steel sample cup and loaded into the VSA, which is between the 0.5 to 5 g recommendation for the equipment. The duration of the experiments varied from 2 to 4 d depending on the set temperature, with higher temperatures concluding more quickly than lower temperatures. Upon completion, the data from the experiments were saved in an Excel file (Microsoft, Redmond, WA).

Experiments were designed as a randomized complete block, and isotherm data were analyzed with ANOVA using JMP Pro 15 (SAS Institute Inc., Cary, NC) to evaluate the relationship between changing temperatures and milk powder type on the moisture sorption isotherms. For the ANOVA, adsorption and desorption equilibrium moisture contents (**EMC**) served as the response variables, which were analyzed individually, with temperature, ERH, and milk powder type as experimental factors. For this test, statistically significant differences were defined by *P* < 0.05. To check for any hysteresis in the adsorption and desorption isotherms, matched-pairs tests were conducted.

The data for NFDM and MPC-85 were fit to several mathematical models for predicting adsorption and desorption EMC, all of which are approved by the American Society of Agricultural and Biological Engineers (modified Halsey, modified Henderson, modified Oswin, and modified Chung-Pfost models, and Guggenheim, Anderson, de Boer [**GAB**] equation). These equations serve as standard equations used for defining sorption isotherms (ASAE, 2002). A list of these model equations can be found in Table 1. These models were selected for the present study due to their use in other studies examining the isotherms of similar products (Kumar et al., 2012).

**Table 1:** Prediction models of equilibrium moisture content (EMC) using temperature and water activity

Equation	EMC model^1^	Reference
Modified Chung-Pfost	$M_{e}=\frac{1}{C}ln\left(\left(\frac{B\;-T}{A}\right)lna_{w}\right)$	Chung and Pfost, 1967
Modified Henderson	Me=-ln1-awAT+B11CC	[Bibr bib12][Bibr bib34]
Modified Halsey	Me=-expA+B×Tlnaw11CC	Iglesias and Chirife, 1976a,b
Modified Oswin	Me=A+BTaw1-aw11CC	[Bibr bib26][Bibr bib7]
GAB	Me=ABCCTTaw1-Baw1-Baw+CCTTBaw	[Bibr bib37][Bibr bib27]

1 *T* is temperature (°C), *a_w_* is water activity in decimal (ERH = *a_w_* × 100), *M_e_* is the equilibrium moisture content (%, dry basis), and *A*, *B*, and *C* are equation or model coefficients. Original references should be consulted before using the equations as listed here.

To find the model coefficients, a nonlinear regression was used (JMP Pro 15). To evaluate the goodness of fit of the models to the data, root mean square error (**RMSE**) and χ^2^ were calculated to determine the goodness of fit for each model. Lower values of RMSE and χ^2^ indicated better fit (Chapra and Canale, 2010).

The moisture sorption isotherms for NFDM and MPC-85 are shown in Figure 1. The NFDM adsorption isotherm exhibited the characteristic sigmoid isotherm shape (type II; Figure 1A) due to a concurrent increase in the moisture content with an increase in ERH, indicating a clear transition point, as outlined in Brunauer et al. (1940). However, NFDM desorption and MPC-85 adsorption and desorption all displayed J-shaped (type III) isotherms (Figures 1B–D), which is characteristic of materials with crystalline components (Labuza and Altunakar, 2007). Materials demonstrating type III isotherm behavior exhibit small gains in moisture up to a certain point (usually a_w_ 0.70–0.80), which is when crystalline components begin to dissolve in the adsorbed water. This trend was shown in the NFDM desorption isotherm and the MPC-85 adsorption and desorption isotherms. Overall, the moisture contents increased with increasing a_w_. However, the moisture contents for both powders increased more rapidly at 60°C compared with 23°C and 40°C, which showed more gradual increases. At 40°C, the moisture content of both powders began to increase rapidly and sharply after a_w_ 0.30 until reaching a_w_ 0.50. Furthermore, at 60°C for both powders, the moisture contents increased rapidly between a_w_ 0.20 to 0.50. The observed trends may be a result of capillary condensation, in which adsorption from the vapor phase into a porous medium continues until the pores are filled with liquid (Tham et al., 2016). As the NFDM and MPC-85 began to adsorb moisture, the powder shifted to both a crystalline and an amorphous state. For powders that contain a mixture of both crystalline and amorphous components, greater water sorption capacity has been observed compared with pure powder (Hartmann and Palzer, 2011). As a result of this compositional change in NFDM and MPC-85, higher and more rapid moisture adsorption was observed after the initial moisture uptakes at a_w_ 0.45, 0.30, and 0.20 for 23°C, 40°C, and 60°C, respectively. The glass transition point decreased with increased temperature, and the higher-lactose product (NFDM) displayed this point at lower relative humidity or moisture content.

**Figure 1:**
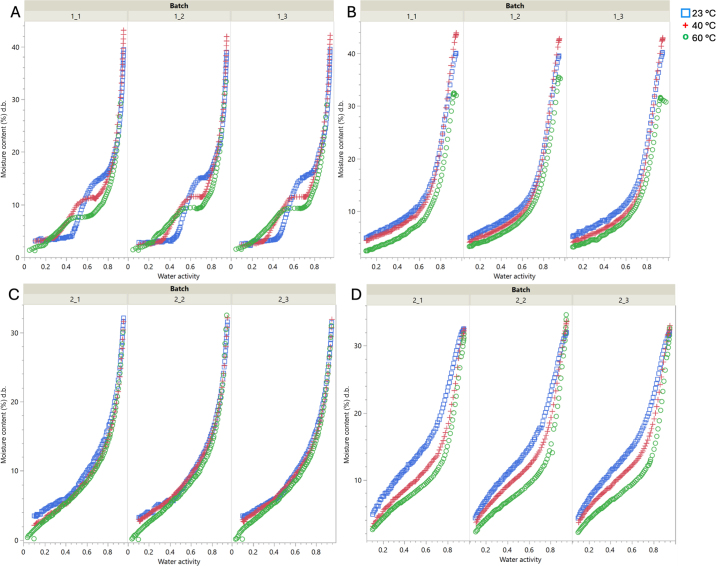
Isotherms of milk powder adsorption (A), nonfat milk powder desorption (B), milk protein concentrate with 85% protein (MPC-85) adsorption (C), and MPC-85 desorption (D) at 23°C, 40°C, and 60°C in 3 independent experiments. Moisture content (% dry basis [d.b.]) as a function of water activity.

Using ANOVA to evaluate the effect of temperature and sample type on moisture sorption isotherms, specific comparisons were made between the milk powders. Temperature had a significant effect on both the adsorption and desorption isotherms (*P* < 0.0001) for both NFDM and MPC-85. The results of an ANOVA conducted for all adsorption across both powders showed that the different replicates (1–3) for each powder were significantly different from one another (*P* = 0.0009), but this difference between replicates was not found for all desorption (*P* = 0.9931). Furthermore, the results of another ANOVA showed the significance of temperature on NFDM adsorption and desorption isotherms (*P* < 0.0001). Other ANOVA results also showed the significance of temperature on both MPC-85 adsorption and desorption isotherms (*P* < 0.0001). A matched-pair analysis revealed hysteresis between the adsorption and desorption curves for both milk powders for all temperatures tested. In other words, at a given a_w_, the desorption EMC was higher than the adsorption EMC (i.e., desorption curves were higher than adsorption curves for all powders, batches [replications], and temperatures). Notably, MPC-85 exhibited greater hysteresis than NFDM for all batches and temperatures, which was unexpected considering its low lactose content, but is important for quality considerations.

The adsorption and desorption isotherms for NFDM and MPC-85 were fitted to the modified Henderson, modified Oswin, modified Halsey, modified Chung-Pfost, and GAB equations (Table 1). The resulting values of coefficients and goodness of fit are shown in Table 2. These models have been used for a variety of other low-a_w_ foods, such as milk powders, cereal grains, and hemp flour (Chung and Pfost, 1967; Štencl, 1999; Luthra et al., 2020; Oduola et al., 2022). Of the models tested for NFDM adsorption, the modified Oswin model had the best fit for 2 out of the 3 replicates except for replicate 1, where the modified Halsey model had the best fit. However, the difference in goodness of fit (RMSE and χ^2^) was very minimal between the modified Halsey versus modified Oswin models for replicate. For NFDM desorption, the GAB equation was found to be the best fit for all 3 replicates (RMSE 1.41–1.49; χ^2^ 1.43–2.22). Notably, when looking at the MPC-85 modeled isotherms, for both the adsorption and desorption isotherms, the RMSE and χ^2^ values were considerably lower (indicative of good fit) compared with the NFDM adsorption and desorption models, suggesting predictability may vary by material. For MPC-85 adsorption, the GAB equation was the most suitable model for all replicates (RMSE 0.30–0.40; χ^2^ 0.12–0.16), whereas the modified Chung-Pfost model was the most suitable for the MPC-85 desorption isotherms for all replicates (RMSE 0.79–0.90; χ^2^ 0.73–0.84). Foster et al. (2005) also found the GAB model to adequately describe desorption for milk powders (skim and whole milk) across a large temperature range, but the study did not investigate adsorption. The GAB model parameters determined by Bronlund and Paterson (2004) of amorphous lactose were also similar to those in the present study, indicating consistency in using the GAB model for milk powder varieties.

**Table 2:** Average estimated model coefficients with RMSE and χ^2^ values fitted to adsorption and desorption data measured for 3 batches at 3 different temperatures (23°C, 40°C, and 60°C)^1^

Isotherm	Model	*A*	*B*	*C*	RMSE	χ^2^
NFDM adsorption	Modified Chung-Pfost	436.84	−210.31	−0.11	3.16	10.03
	Modified Henderson	0.00042	296.87	0.85	2.15	4.67
	Modified Halsey	2.59	−0.0024	1.46	1.74	3.04
	Modified Oswin	7.65	−0.016	1.69	1.67	2.76
	GAB	3.89	0.95	432.42	1.83	3.43
NFDM desorption	Modified Chung-Pfost	234.59	−53.86	−0.14	1.96	3.93
	Modified Henderson	0.0004	129.77	1.2	1.61	2.62
	Modified Halsey	4.17	−0.01	1.94	1.78	3.23
	Modified Oswin	10.31	−0.05	2.3	1.57	2.51
	GAB	5.93	0.85	199.3	1.36	1.90
MPC-85 adsorption	Modified Chung-Pfost	511.59	−154.2	−0.18	0.93	0.87
	Modified Henderson	0.0001	326.87	1.2	0.66	0.44
	Modified Halsey	3.28	−0.003	1.85	0.85	0.73
	Modified Oswin	7.34	−0.012	2.19	0.49	0.24
	GAB	4.33	0.87	340.53	0.35	0.14
MPC-85 desorption	Modified Chung-Pfost	223.84	−19.45	−0.18	0.85	0.8
	Modified Henderson	0.0002	51.75	1.60	1.13	1.31
	Modified Halsey	5.65	−0.017	2.4	1.41	1.99
	Modified Oswin	12.18	−0.065	2.86	1.12	1.26
	GAB	8.59	0.71	185.0	1.15	1.32

1 *A*, *B*, and *C* are equation or model coefficients from original references described in Table 1.

The results presented illustrate the unique sorption trends of NFDM and MPC-85, 2 distinctly different milk powders, which the DDI method uniquely captured with accuracy due to its capabilities (Meter Group, 2022). Although both powders were of bovine milk origin, their individual compositions varied greatly, which may contribute to the variable isotherm shape. Several studies examine the moisture sorption isotherms of a variety of milk powders and what factors may be responsible for the sorption behavior observed between different milk powders, such as protein concentration, temperature, and the presence of crystalline lactose (Bronlund and Paterson, 2004; Foster et al., 2005; Shrestha et al., 2007; Kumar et al., 2012). Hssaini et al. (2022) also found temperature to have a significant effect in the case of fig, and effects on isotherms have also been noted due to product and sample composition (Rodríguez-Martínez et al., 2020). The presence of high concentration of protein in the MPC-85 sample may have contributed to its adsorption (type III) compared with NFDM adsorption (type II). The protein content of milk may affect change of state from crystalline to amorphous and vice versa, which may in turn influence the water adsorption rate (Foster et al., 2005; Labuza and Altunakar, 2007).

To reflect conditions powders may encounter during processing and storage, sorption isotherms were measured at 23°C, 40°C, and 60°C. The 23°C data represented ambient, climate-controlled environments, whereas 40°C simulated uncontrolled warehouse conditions. The 60°C condition was included due to its proximity to protein denaturation temperatures (>65°C; Wagner et al., 2020). Adsorption and desorption differed between NFDM and MPC-85, likely due to their distinct protein contents (NFDM: 37.59% ± 0.65%; MPC-85: 86.38% ± 0.17%). Higher protein levels promote greater moisture adsorption due to increased water-binding capacity (Shrestha et al., 2007), which was reflected in the results of this study.

Thermal treatments may be applied to milk powders by a manufacturer if they require a preventive control suitable to inactivate pathogens such as *Salmonella*, which was one of the driving applications of the present study. Although thermal treatment temperatures effective against *Salmonella* have been found to be greater than 60°C (75°C, 80°C, and 85°C; Kadas, 2022), 60°C was the highest test parameter for temperature within the capabilities of the VSA. At 60°C, some heat-induced protein denaturation was expected in a temperature range where protein aggregation and denaturation are typically observed for kinds of whey protein at certain elevated temperatures. Li et al. (2021) noted that β-LG and α-LA denature between 70°C and 75°C, but BSA and lactoferrin are more sensitive, denaturing around 65°C. This change in protein structure and content is an important consideration for the dairy industry as they look to implement their own thermal process based on the results presented in this work. For both NFDM and MPC-85, the powders were yellowed upon completion of the 60°C isotherms, indicating that processors may not want to treat dairy powders at significantly higher temperatures if quality degradation would be problematic. Yellowing of the powders was also observed during the thermal treatments at each temperature. The color change is also an important consideration for the dairy industry when assessing acceptability.

Processors tend to target a_w_ ≤0.65 in dry foods, as water activity at or below this point halts microbial growth, both bacterial and fungal. Moisture sorption isotherms are not a metric for microbiological safety, but they link a_w_ and moisture content trends. Most processors use moisture content over a_w_ when testing their products, because it is much easier (and less expensive) to measure moisture content. From moisture content, isotherms can be used to determine which a_w_ (at a set temperature) corresponds to a specified moisture content, and then decisions can be made about packaging, shelf life, and product stability.

Moisture sorption isotherms for NFDM and MPC-85 revealed how temperature and composition affect moisture movement in milk powders. These results emphasize the need to consider storage conditions and formulation when predicting quality and shelf life. In particular, NFDM requires careful, low-humidity storage due to its higher lactose content, lower glass transition point, and tendency to absorb moisture and clump.
